# Prognoses of Patients with Hormone Receptor-Positive and Human Epidermal Growth Factor Receptor 2-Negative Breast Cancer Receiving Neoadjuvant Chemotherapy before Surgery: A Retrospective Analysis

**DOI:** 10.3390/cancers15041157

**Published:** 2023-02-10

**Authors:** Shichao Zhang, Yan Liu, Xu Liu, Yingxue Liu, Jin Zhang

**Affiliations:** The 3rd Department of Breast Cancer, Tianjin Medical University Cancer Institute and Hospital, National Clinical Research Center for Cancer, Key Laboratory of Cancer Prevention and Therapy, Tianjin, Tianjin’s Clinical Research Center for Cancer, Key Laboratory of Breast Cancer Prevention and Therapy, Tianjin Medical University, Ministry of Education, Tianjin 300060, China

**Keywords:** breast cancer, neoadjuvant chemotherapy, survival, prognosis, PR, HER2-low

## Abstract

**Simple Summary:**

To date there is no specific analysis providing comprehensive available evidence that can guide clinical treatment options for patients with hormone receptor-positive and human epidermal growth factor receptor-2-negative (HR+/HER2−) breast cancer (BC) after neoadjuvant chemotherapy (NAC). We conducted a retrospective study with 3070 consecutive HR+/HER2− BC patients after NAC and indicated that progesterone receptor (PR) negativity BC has a higher complete pathological response (pCR) and lower survival than PR positivity BC. PR may be used as an independent predictor for pCR and a prognosis of HR+/HER2− BC in the NAC setting. HER2-low tumors are not significantly associated with pCR or disease-free survival (DFS), but these patients have significantly lower overall survival (OS) than those with HER-0 tumors. Our research confirms the significance of PR and HER2 status associated with pCR and prognosis.

**Abstract:**

Purpose: To evaluate the clinical characteristics, pathological response, and prognostic significance of hormone receptor-positive and human epidermal growth factor receptor 2-negative (HR+/HER2−) breast cancer (BC) after neoadjuvant chemotherapy (NAC). Methods: A survival analysis was performed to detect the factors related to recurrence and death in 3070 consecutive patients with HR+/HER2− BC who received NAC from 2011 to 2022. All patients received current “standard of care” following neoadjuvant therapy based on guidelines, including surgery and adjuvant endocrine therapy. HER2-low was defined as immunohistochemistry (IHC) 1+ or IHC 2+ and fluorescence in-situ hybridization-negative. Results: The complete pathological response (pCR) (ypT0/is ypN0) rate was 14.5%. The clinical tumor size (cT), ER scores, PR status, and Ki67 levels were related to pCR. The 5-year disease-free survival (DFS) and overall survival (OS) rates were 82.6% and 90.4%, respectively. PR, Ki67 levels, and postmastectomy radiotherapy were independent factors for DFS and OS, and the extranodal extension (ENE) correlated with DFS. However, pCR and HER2 status were related to OS. The pCR rate in PR negativity BC was significantly higher than that in PR positivity BC (21.1% vs. 12.2%, *p* = 0.000), but PR negativity BC had a poorer prognosis than PR positivity BC. HER2-low BC showed high ER scores (over 50%), PR positivity, large ypT, ENE, and lymphovascular invasion but a lower pCR rate than HER2-zero BC. Patients with HER2-low BC had shorter OS than those with HER2-zero BC (*p* = 0.037). However, there was no difference in DFS. Conclusions: Depending on PR status and HER2 status, patients with ER positivity and HER2 negativity exhibit different pathologic complete response rates to neoadjuvant chemotherapy and long-term outcomes, especially patients with PR negativity or HER2-low status.

## 1. Introduction

In 2020, breast cancer (BC) has already exceeded lung cancer as the leading cause of worldwide cancer incidence, accounting for 11.7% of all cancer cases [1], and the incidence and mortality of BC have also been increasing in China over the past several years [2]. The hormone receptor-positive and human epidermal growth factor receptor 2-Negative (HR+/HER2−) subtype, accounting for approximately half of BC cases in China [3], is the most common molecular subtype [4]. In the next three years, its incidence rate will increase by 2% yearly [5,6,7]. Although tailored anti-estrogen drugs for estrogen receptor (ER)-positive BC have been applied in adjuvant therapy, 30–50% of BC patients will experience relapse due to primary or acquired resistance to drugs [8]. A recent study with 1848 patients reported that disease progression occurs in over 60% of patients with advanced HR+/HER2− BC [9].

For locally advanced and inoperable BC, neoadjuvant chemotherapy (NAC) is the current standardized treatment [10]; achieving a complete pathological response (pCR) leads to a favorable prognosis [11]. Specifically, patients with HR+/HER2− BC are less likely to achieve pCR than those with triple-negative BC (TNBC) or HER2+ BC [12,13,14]. A pooled analysis of 12 randomized trials with approximately 2000 HR+/HER2− BC patients showed that pCR was significantly associated with event-free survival (EFS). However, this trend was primarily observed in poorly differentiated tumors [11]. Another analysis of 8244 patients with HR+/HER− BC showed that the pCR rate was 8.1% and that pCR was significantly linked to 5-year overall survival (OS) [15]. However, the predictors for pCR were also inconsistent across the above studies. Moreover, some studies have noted that residual disease after NAC does not necessarily result in a worse prognosis for the HR+/HER2− BC subtype [11,16]. In general, it is necessary to find reliable prognostic factors to identify HR+/HER2− BC patients after NAC with recurrence risk and provide individualized adjuvant treatment. Although several researchers have reached conflicting conclusions about prognostic factors for HR+/HER2− BC [17,18], a comprehensive summary and evaluation of these poor prognostic factors are still lacking.

This study aimed to evaluate the clinicopathological characteristics, pCR, and prognosis of HR+/HER2− BC patients in the NAC setting.

## 2. Materials and Methods

### 2.1. Patients

Patients with HR+/HER2− BC treated with NAC who were hospitalized at the Tianjin Medical University Cancer Institute and Hospital from January 2011 to January 2022 were included in this retrospective study. Only HR+ and HER2− BC female patients without distant metastasis at initial diagnosis were included. The main selection criteria included (1) pathologically diagnosed invasive ductal breast carcinoma; (2) HR-positive by immunohistochemistry (IHC) and HER2-negative by IHC or fluorescence in situ hybridization (FISH); (3) received NAC; and (4) underwent surgery followed NAC. The main exclusion criteria included (1) no surgery was performed followed NAC and efficacy of NAC could not be assessed based on pathology; and (2) follow-up data were incomplete.

Clinical information was collected, including age, menopausal status, family history of BC, clinical TNM stage, cancer recurrence details, and survival status. Pathological information was also collected, including IHC results (ER, PR, HER2, Ki67, p53), pathological TNM stage, and response to NAC. According to St. Gallen guidelines 2013 [19], 14% was adopted as the cutoff value of Ki67 for classification. In reference to ASCO guidelines [20], 1% was set as the cutoff index between “negative” and “positive” for PR, and HER2 status was strictly graded as follows: 0, 1+, 2+, and 3+. HER2(−) status was defined as 0, 1+, and 2+ with negative FISH results. To ensure accuracy, all pathology specimens of the patients were reviewed by two experienced pathologists. The deadline for follow-up was September 2022. Disease-free survival (DFS) was defined as the date of surgery to the first recurrence event (local-regional recurrence or distant metastasis), and OS was calculated from the date of surgery to cancer-related death or the last follow-up.

### 2.2. Statistical Analysis

The chi-square test was performed to identify discrepancies in variable distributions. The log-rank test was performed to investigate the association between univariate factors and survival time, and Kaplan–Meier curves were utilized to visualize the results of the survival analysis. The Cox proportional hazards regression model was used to identify significant independent risk factors related to DFS and OS for overall patients; multivariable binary logistic regression analysis was used to identify prognostic factors predicting pCR and factors assciated with HER2 status. The SPSS 25.0 software (IBM., Armonk, NY, USA) was employed for all statistical analyses. In all studies, a *p* value < 0.05 was defined as statistically significant.

## 3. Results

### 3.1. Patient and Treatment Characteristics

**Figure 1:**
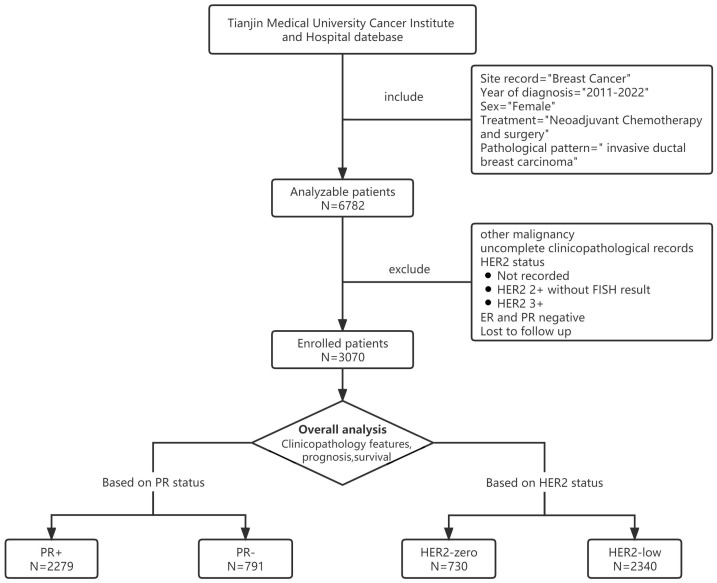
Patient selection flowchart for this study.

In total, 3070 consecutive patients with HR+/HER2− BC after NAC were included in this study. Patients with unknown HER2 status (n = 48) and those who missed follow-up (n = 55) were excluded (Figure 1). The baseline characteristics of the patients are shown in Table 1. The 3070 patients were all female, with a median age of 50 years (range: 22–85 years). The proportions of premenopausal and postmenopausal women were 59.7% and 40.3%, respectively. Most tumors were ypT2 (44.1%) and ypN1-3 (69.9%). A total of 87.3% of patients had ER scores over 50%, and 74.2% of BCs were PR positivity. The rates of extranodal extension (ENE) and lymphovascular invasion were 34.1% and 26.3%, respectively. All patients received a minimum of two cycles of the NAC regimen, including taxane and anthracycline. After surgery, all patients received adjuvant endocrine therapy and regimens, including aromatase inhibitors (AIs) and selective estrogen receptor modulators (SERMs). Ovarian function suppression (OFS) was routinely adopted for premenopausal women. Radiotherapy was required in cases of cT3-T4 or nodal involvement before NAC or ypN+ tumors; 69.1% of the patients in this cohort received radiotherapy. A total of 577 patients (18.8%) experienced a recurrence or metastasis, and 361 patients (11.7%) died due to BC.

### 3.2. Follow-Up and Survival

Following a median follow-up period of 40 months (range from 2 to 141 months), the 5-year DFS and OS rates were 82.6% and 90.4%, respectively. As displayed in Table 1, univariate analysis showed that ypT, ypN, PR, Ki67 level, radiotherapy, endocrine therapy, and ENE were significant prognostic factors for DFS. Multivariate Cox regression analysis (shown in Table 2) identified PR (0.730, 95% CI: 0.606–0.881, *p* = 0.001), radiotherapy (0.537, 95% CI: 0.433–0.665, *p* = 0.000), Ki67 level (1.399, 95% CI: 1.181–1.656, *p* = 0.000), and ENE (1.298, 95% CI: 1.072–1.570, *p* = 0.007) as independent prognostic factors for DFS. ypT, ypN, ER score, PR, HER2 status, Ki67 level, pCR, radiotherapy, and ENE were significant prognostic factors for OS in univariate analysis (Table 1), and radiotherapy (0.592, 95% CI: 0.452–0.774, *p* = 0.000), PR (0.486, 95% CI: 0.380–0.621, *p* = 0.000), Ki67 levels (1.635, 95% CI: 1.310–2.042, *p* = 0.000), and HER2 status (1.385, 95% CI: 1.051–1.826, *p* = 0.040) were independent prognostic factors for OS in multivariate analysis (Table 2). Notably, PR and Ki67 levels were independent prognostic factors for DFS and OS in this cohort; HER2 status was an independent significant prognostic factor only for OS but not for DFS.

### 3.3. Pathological Response to NAC

**Figure 2:**
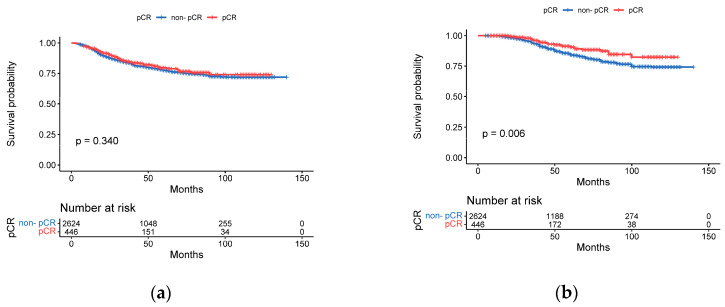
Kaplan–Meier curves for disease-free survival (DFS) and overall survival (OS) by pCR. (**a**) DFS for non-pCR vs. pCR (**b**) OS for non-pCR vs. pCR.

The pCR rate of the cohort was 14.5%. As shown in Table 3, we subdivided patients into two subgroups according to their pathological response to NAC. The univariate analysis showed that these clinicopathologic factors, including age, menopausal status, cT, cN, ER score, PR, HER2, and Ki67 level, were associated with pCR. Next, a binary logistic regression analysis was conducted to evaluate the associated factors, which showed that cT (0.723, 95%, CI: 0.607–0.862, *p* = 0.000), ER score (0.713, 95% CI: 0.636–0.798, *p* = 0.000), PR (0.644, 95% CI: 0.515–0.806, *p* = 0.000), and Ki67 levels (0.490, 95% CI: 0.394–0.608, *p* = 0.000) were significant independent factors of pCR. Although the distribution of HER2-low was statistically significant between the two subgroups (77.0% vs. 71.7%, *p* = 0.016), HER2-low had no predictive value for pCR (0.800, 95% CI: 0.635–1.009, *p* = 0.059). pCR was significantly associated with OS between the two subgroups (*p* = 0.006) (Figure 2b) but with no significant difference in DFS (*p* = 0.340) (Figure 2a).

### 3.4. Disease Outcomes in PR Negativity vs. PR Positivity Subgroups

#### 3.4.1. Comparison of Clinicopathological Characteristics by PR Status

As shown in Table 4, compared with the PR positivity subgroup, more patients in the PR negativity subgroup showed ypT0 (22.4% vs. 13.6%, *p* = 0.000), ypN0 (35.7% vs. 28.1%, *p* = 0.000) and higher pCR (21.1% vs. 12.2%, *p* = 0.000). However, fewer PR negativity patients showed Ki67 > 14% (47.0% vs. 55.8%, *p* = 0.000), ENE positivity (30.0% vs. 35.6%, *p* = 0.004), ER scores > 50% (72.4% vs. 92.4%, *p* = 0.000), and P53 positivity (49.7% vs. 61.6%, *p* = 0.000). No statistically significant difference was found in age, family history, menopausal status, radiotherapy, endocrine therapy, or lymphovascular invasion between these two groups.

#### 3.4.2. Comparison of DFS and OS by PR Status

**Figure 3:**
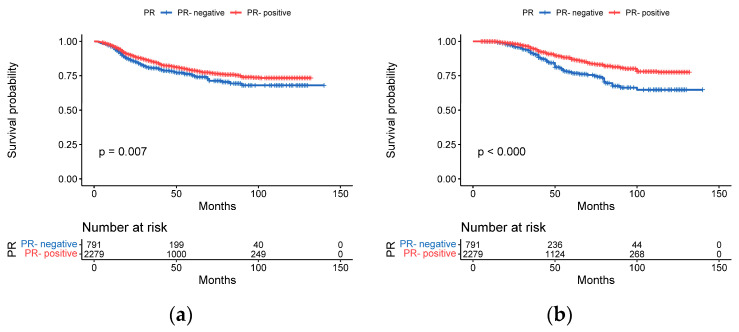
Kaplan–Meier curves for DFS and OS by PR status. (**a**) DFS for PR negativity vs. PR positivity (**b**) OS for PR negativity vs. PR positivity.

The DFS rates of the PR negativity and PR positivity subgroups were 80.5% and 81.3%, respectively, and the OS rates were 86.6% and 88.8%, respectively. Patients in the PR negativity subgroup experienced more recurrence than those in the PR positivity subgroup (*p* = 0.007, Figure 3a). Patients in the PR negativity subgroup survived for a significantly shorter period of time than those in the PR positivity subgroup (*p* < 0.001, Figure 3b).

### 3.5. Disease Outcomes in HER2-Zero vs. HER2-Low Tumors

#### 3.5.1. Comparison of Clinicopathological Characteristics by HER2 Status

Table 5 lists the baseline clinicopathological features according to HER2 status. Of the 3070 patients recruited, 2340 (76.2%) had HER2-low tumors. Compared with HER2-zero patients, more HER2-low patients showed PR positivity (75.5% vs. 70.1%, *p* = 0.004), ENE positivity (35.3% vs. 30.3%, *p* = 0.012), lymphovascular invasion (27.9% vs. 21.1%, *p* = 0.000), and P53 positivity (60.5% vs. 52.2%, *p* = 0.000), and fewer patients achieved pCR (13.7% vs. 17.3%, *p* = 0.016). More patients with HER2-low tumors were classified as ER ≥50% (88.3% vs. 84.1%, *p* = 0.001) and high ypT (T1-4: 85.2% vs. 80.7%, *p* = 0.001) compared with the HER2-zero subgroup. No statistically significant difference was found in age, family history, menopausal status, stage N, or Ki67 status between these two groups. Binary logistic regression (Table 6) was conducted to analyze the relationship between HER2 status and statistically significant factors identified by univariate analysis, showing that the pCR status, ypT, and PR were not significantly linked to HER2 status. The ER score (1.327, 95% CI: 1.197–1.472, *p* = 0.000), P53 (1.381, 95% CI: 1.164–1.637, *p* = 0.000), ENE (1.233, 95% CI: 1.020–1.489, *p* = 0.030), and lymphovascular invasion (1.431, 95% CI: 1.168–1.752, *p* = 0.001) were statistically significant factors associated with HER2 status.

#### 3.5.2. Comparison of DFS and OS by HER2 Status

**Figure 4:**
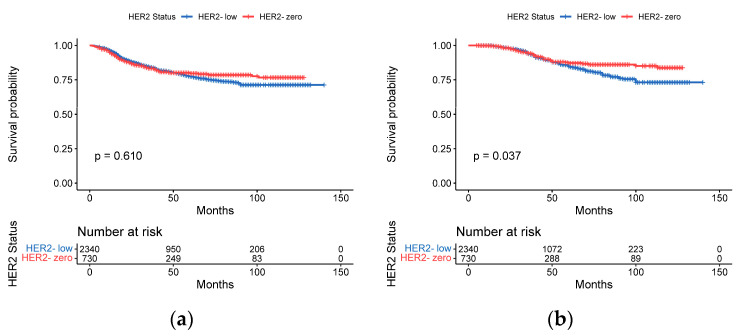
Kaplan–Meier curves for DFS and OS by HER2 status. (**a**) DFS for HER2-zero vs. HER-low (**b**) OS for HER2-zero vs. HER-low.

We also analyzed DFS and OS by painting Kaplan–Meier curves to determine the effect of HER2 status on survival. The DFS rates of the HER2-low and HER2-zero subgroups were 80.7% and 82.9%, respectively. However, no significant difference in DFS was found between these two subgroups (*p* = 0.61; Figure 4a). The OS of the HER2-low and HER2-zero subgroups was 87.3% and 91.4%, respectively, and patients in the HER2-low subgroup survived for a significantly shorter period of time than those in the HER2-zero subgroup (*p* = 0.037, Figure 4b).

## 4. Discussion

Although the CTNeoBC pooled analysis has showed HR+/HER2− BC patients with a high grade who attained pCR could improve survival [21], the results of further subgroup analysis of HR+/HER2− BC patients were not yet reported. To the best of our knowledge, this retrospective study is the first and largest cohort of HR+/HER2− BC patients after NAC to evaluate factors affecting the prognosis and identify predictors of the pCR in China.

The pCR rate of this cohort was only 14.5%, which was similar to the range of 7.5% to 16.2% in a previous study [11]. ER-positive BC is thought to be less susceptible to chemotherapy than other subtypes [11], which may indicate that additional neoadjuvant endocrine therapy (NET) will be beneficial. A retrospective study reported that HR+/HER2− tumors treated with NET (n = 127) or NAC (n = 338) reached similar ratios of nodal pCR (11% vs. 18%, respectively), which means that NET may have an effect equivalent to that of NCT [22]. Moreover, our study showed that pCR was significantly associated with OS (*p* = 0.006) but not DFS, which agrees with a previous study [15]. Reasonable neoadjuvant therapy, including neoadjuvant endocrine therapy, can improve the pCR rate and prolong survival to some extent.

Additionally, we found that PR, Ki-67 level (14% in this study), ENE, and postmastectomy radiotherapy were significant independent factors for DFS and that PR, Ki-67 level, HER2, and postmastectomy radiotherapy were related to OS.

Several studies have proven the correlation between PR levels and the Oncotype DX recurrence score (RS), showing that combining the PR and mitotic rate can serve as a substitute index for Oncotype RS [23,24,25]. According to our results, although PR negativity tumors displayed higher pCR, more Ki67 levels <14%, and lower rates of ENE, they still had a poorer prognosis than PR positivity tumors. A previous study indicated that reduced PR levels might explain the different prognoses and specific ER modulator resistances in patients with ER+/PR– BC [26]. As the final product of ER metabolism, the PR level is usually thought to depend on ER activity. Thus, a decrease in PR expression reflects a hypofunctional status of ER and resistance to endocrine drugs [27,28]. Bardou et al. found that PR status is an independent factor in predicting the benefit of adjuvant anti-estrogen therapy [29]. In our study, PR status was associated with ER scores, and PR negativity tumors showed lower ER scores. PR negativity BC showed lower DFS and OS, indicating that PR status may be related to the effectiveness of endocrine therapy. However, a review of the ATAC and BIG 1–98 trials found that PR levels did not affect the survival benefits of adjuvant endocrine therapy [30,31]. More clinical studies are needed to determine the relationship between PR status, endocrine therapy resistance, and prognosis in HR+/HER2− BC patients.

Since Cheang et al. reported that Ki67 effectively distinguishes Luminal A from Luminal B subtypes [32,33], the critical value of Ki67 in BC has been intensively explored through clinical research but is still under debate. Many studies have demonstrated that a high Ki67 index is associated with poor prognosis [10,34]. Furthermore, some studies have noted that the Ki67 index of residual tumors is predictive of long-term outcomes for patients after NAC [33,34]. In this study, we used 20% as the cutoff value for Ki67, but no signs related to prognosis were found (data not shown). Therefore, we adopted the proposal of the 2013 St. Gallen consensus statement, setting 14% as the cutoff value for dichotomous classification of Ki67 [11] and identifying its significance for prognosis. This may indicate that for HR+ and HER2− patients after NAC, the low cutoff value of 14% might reflect the true efficacy of NAC to some extent. Nevertheless, few studies have examined the Ki67 index as a continuous variable to explore its clinical significance, and additional research is needed.

In this study, postmastectomy radiotherapy was a significant independent factor for DFS and OS. Patients with a significant residual tumor burden, such as lymph node metastasis or ypN2/3 status, derive a survival advantage from postmastectomy radiotherapy [35]. Additionally, JC Ma et al. [36] proposed a new prognostic nomogram based on 1118 BC patients after NAC, revealing that locoregional recurrence control is improved after radiotherapy in the HR+, HER2− BC subtype, which was supported by our study’s findings.

The introduction of innovative anti-HER2 antibody–drug conjugates, such as trastuzumab deruxtecan, has boosted interest in HER2-low BC in recent years. Our study showed that patients in the HER2-low subgroup had a lower OS rate than those in the HER2-zero subgroup (87.3% vs. 91.4%, *p* = 0.037). However, the impact of HER2-low status on prognosis is still under debate. Chen M et al. [35] found no statistically significant difference in survival outcomes between HER2-low and HER2-zero among HR+ patients. Other studies have compared HER2-zero/HER2 1+ with HER2 2+/ISH-negative patients and noted that the latter had a higher risk of worse outcomes [37,38]. Further analysis showed that disparities were more likely in the HR-positive subgroup than in the HR-negative subgroup [39].

We also found that more cases of HER2-low tumors were classified as ER ≥50% (88.3% vs. 84.1%, *p* = 0.001) than cases of HER2-zero tumors, which agrees with the findings of earlier studies [40,41]. In the present study, pCR rates differed significantly between the HER2-low and HER2-zero subgroups (17.3% vs. 13.7%, *p* = 0.016). In a cohort encompassing 63.4% of patients with HR+/HER2− BC, Moura Leite et al. [42] found that the difference between the pCR rates of HER2-low tumors and HER2-zero tumors was not statistically significant at 13% vs. 9.5%, respectively, with no predictive value for pCR after standard NAC. Diverging from these data, Denkert et al. reported lower pCR rates in the HR+ HER2-low group than in the HR+ HER2-zero group (17.5% vs. 23.6%, *p* = 0.024) when studying 2310 patients in neoadjuvant clinical trials [43]). The reason for our different conclusions may be that not all patients recruited in the studies above received NAC, and that not all studies stratified patients with specific HR+ subtypes for analysis; all of the subjects in our research had HR+ and HER2− BC and underwent NAC. CDK4/6 inhibitors were not used in our cohort for adjuvant endocrine therapy or NET. More studies are needed to determine whether the prognosis is related to HER2 status for HR+/HER2− patients receiving CDK4/6 inhibitors.

We identified ENE as a significant independent factor for DFS. Fisher et al. [44] showed that ENE results in a worse prognosis, which agrees with our findings. Recently, according to the new CAP guidelines, the size/extent of ENE must be reported in pathology papers because it seems to reflect axillary nodal status or poor prognosis. Although ENE, which is significantly associated with DFS, is not utilized to guide treatment decisions, accompanying treatment and enhanced follow-up for patients with ENE may be beneficial. In our study, pN revealed the status of axillary lymph nodes, but it showed no prognostic value, which may be explained by the low proportion of pN0 at 30%.

It should be noted that our study has several limitations. Firstly, over the course of 10 years, it is impossible to totally prevent the potential bias of various chemotherapy regimens adopted and the timing of surgeries. Secondly, pathological assessment methods for Ki-67 and HER2 status have been updating in the last decade, potentially leading to diagnosis and selection errors, but two experienced pathologists were required to review slices to avoid these deviations in our study. Additionally, this study was conducted exclusively at our institution, which may result in inevitable patient selection bias. Further multicenter research is needed to verify our findings.

## 5. Conclusions

We reported the clinicopathological characteristics, pCR, and prognosis for HR+/HER2− BC after NAC. PR may be used as an independent predictor for pCR and prognosis of ER+/HER2− BC in the NAC setting. HER2-low tumors are related to poorer OS than HER2-zero tumors, and combined targeted therapy for HER2-low patients should be implemented.

## Figures and Tables

**Table 1:** Baseline patient characteristics and log rank test of DFS and OS.

Variable	Total (n = 3070)	DFS (n = 577)	Events	*p*	OS (n = 361)	Events	*p*
		Events-Free			Events-Free		
Age				0.193			0.492
<50 years	1688 (55.0)	1386 (55.6)	302 (52.3)		1497 (55.3)	191 (52.9)	
≥50 years	1382 (45.0)	1107 (44.4)	275 (47.7)		1212 (44.7)	170 (47.1)	
Menopausal status				0.058			0.201
Pre/Peri-	1833 (59.7)	1509 (60.5)	324 (56.2)		1629 (60.1)	204 (56.5)	
Post-	1237 (40.3)	984 (39.5)	253 (43.8)		1080 (39.9)	157 (43.5)	
Family history				0.120			0.140
No	2443 (79.6)	1987 (79.7)	456 (79.0)		2158 (79.7)	285 (78.9)	
Breast	221 (7.2)	167 (6.7)	54 (9.4)		182 (6.7)	39 (10.8)	
Others	406 (13.2)	339 (13.6)	67 (11.6)		369 (13.6)	37 (10.2)	
ypT				0.000 *			0.001 *
ypT0	487 (15.9)	410 (16.4)	77 (13.4)		454 (16.8)	33 (9.1)	
ypT1	765 (24.9)	652 (26.2)	113 (19.6)		691 (25.5)	74 (20.5)	
ypT2	1353 (44.1)	1085 (43.5)	268 (46.4)		1176 (43.4)	177 (49.0)	
ypT3-4	465 (15.1)	346 (13.9)	119 (20.6)		388 (14.3)	77 (21.3)	
ypN				0.000 *			0.000 *
ypN0	923 (30.1)	786 (31.5)	137 (23.7)		850 (31.4)	73 (20.2)	
ypN1	782 (25.4)	665 (26.7)	117 (20.3)		713 (26.3)	69 (19.1)	
ypN2-3	1365 (44.5)	1042 (41.8)	323 (56.0)		1146 (42.3)	219 (60.7)	
ER				0.230			0.070 *
0–9%	225 (7.3)	175 (7.0)	50 (8.7)		187 (6.9)	38 (10.5)	
10–49%	165 (5.4)	123 (4.9)	42 (7.2)		134 (5.0)	31 (8.6)	
50–89%	1110 (36.2)	880 (35.3)	230 (39.9)		954 (35.2)	156 (43.2)	
90–100%	1570 (51.1)	1315 (52.8)	255 (44.2)		1434 (52.9)	136 (37.7)	
PR				0.007 *			0.000 *
Negativity	791 (25.8)	637 (25.6)	154 (26.7)		685 (25.3)	106 (29.4)	
Positivity	2279 (74.2)	1856(74.4)	423 (73.3)		2024 (74.7)	255 (70.6)	
HER2				0.611			0.037 *
Zero	730 (23.8)	605 (24.3)	125 (21.7)		667 (24.6)	63 (17.5)	
Low	2340 (76.2)	1888 (75.7)	452 (78.3)		2042 (75.4)	298 (82.5)	
Ki67				0.000 *			0.000 *
≤14%	1427 (46.5)	1204 (48.3)	223 (38.6)		1302 (48.1)	125 (34.6)	
>14%	1643 (53.5)	1289 (51.7)	354 (61.4)		1407 (51.9)	236 (65.4)	
P53				0.572			0.322
Negative	1273 (41.5)	1042 (41.8)	231 (40.0)		1138 (42.0)	135 (37.4)	
Positive	1797 (58.5)	1451 (58.2)	346 (60.0)		1571 (58.0)	226 (62.6)	
pCR (ypT0/is, ypN0)				0.344			0.006 *
No	2624 (85.5)	2119 (85.0)	505 (87.5)		2293 (84.6)	331 (91.7)	
Yes	446 (14.5)	374 (15.0)	72 (12.5)		416 (15.4)	30 (8.3)	
Surgery				0.440			0.389
Mastectomy	2694 (87.7)	2303 (85.5)	391 (86.0)		2378 (87.8)	316 (87.5)	
Breast-conserving surgery	376 (12.3)	190 (14.5)	186 (14.0)		331 (12.2)	45 (12.5)	
Radiotherapy				0.000 *			0.000 *
No	948 (30.9)	840 (33.7)	108 (18.7)		877 (32.4)	71 (19.7)	
Yes	2122 (69.1)	1653 (66.3)	469 (81.3)		1832 (67.6)	290 (80.3)	
Endocrine therapy				0.000 *			0.138
AI ± OFS	2372 (77.3)	1901 (76.2)	471 (81.6)		2090 (77.2)	282 (78.1)	
SERM ± OFS	698 (22.7)	592 (23.8)	106 (18.4)		619 (22.8)	79 (21.9)	
Extranodal extension				0.000 *			0.000 *
Yes	1048 (34.1)	783 (31.4)	265 (45.9)		876 (32.3)	172 (47.6)	
No	2022 (65.9)	1710 (68.6)	312 (54.1)		1833 (67.7)	189 (52.4)	
Lymphovascular Invasion				0.146			0.999
Yes	807 (26.3)	639 (25.6)	168 (29.1)		712 (26.3)	95 (26.3)	
No	2263 (73.7)	1854 (74.4)	409 (70.9)		1997 (73.7)	266 (73.7)	

* indicates statistically significant results. ER, estrogen receptor; PR, progesterone receptor; HER2, human epidermal growth factor receptor 2; pCR, pathologic complete response; DFS, disease-free survival; OS, overall survival.

**Table 2:** Multivariate COX regression analysis of overall DFS and OS.

	Multivariate Analysis		
Variables	HR	95%CI	*p*
DFS			
ypT	-	-	0.195
ypT1	0.873	0.626–1.218	0.421
ypT2	1.004	0.732–1.377	0.980
ypT3-4	1.172	0.824–1.666	0.379
ypN	-	-	0.189
ypN1	0.885	0.660–1.186	0.420
ypN2-3	1.098	0.828–1.456	0.518
Radiotherapy	0.537	0.433–0.665	0.000 *
Endocrine therapy	0.851	0.682–1.062	0.151
PR	0.730	0.606–0.881	0.001 *
Ki67	1.399	1.181–1.656	0.000 *
Extranodal extension	1.298	1.072–1.570	0.007 *
OS			
ypT	-	-	0.644
ypT1	1.197	0.424–3.375	0.734
ypT2	1.312	0.471–3.655	0.604
ypT3-4	1.461	0.518–4.118	0.474
ypN	-	-	0.007
ypN1	0.908	0.615–1.342	0.629
ypN2-3	1.42	0.979–2.059	0.065
Radiotherapy	0.592	0.452–0.774	0.000 *
ER	-	-	0.021
10–49%	1.349	0.858–2.119	0.194
50–89%	1.414	0.975–2.051	0.068
90–100%	1.407	1.120–1.768	0.003
PR	0.486	0.380–0.0621	0.000 *
Ki67	1.635	1.310–2.042	0.000 *
Extranodal extension	0.256	0.904–1.459	1.149
HER2	1.385	1.051–1.826	0.040 *
pCR status	0.978	0.326–2.939	0.969

* Indicates statistically significant results.

**Table 3:** Patient characteristics stratified by pCR status (non pCR vs. pCR).

Variable	NonpCR (n = 2624)	pCR (n = 446)	Univariate Analysis		Binary Logistic Regression Analysis	
			χ^2^	*p* Value *	Odds Ratio (95%CI)	*p* Value *
Age			17.619	0.000 *	0.758 (0.563–1.013)	0.061
<50 years	1402 (53.4)	286 (64.1)				
≥50 years	1222 (46.6)	160 (35.9)				
Menopausal status			29.237	0.000 *	1.023 (0.762–1.375)	0.877
Pre/Peri-	1535 (58.5)	298 (66.8)				
Post-	1089 (41.5)	148 (33.2)				
cT			29.315	0.000 *	0.723 (0.607–0.862)	0.000 *
cT1	199 (7.6)	48 (10.8)				
cT2	1549 (59.0)	280 (62.7)				
cT3-4	876 (33.4)	118 (26.5)				
cN			18.782	0.005 *	1.123 (0.975–1.293)	0.107
cN0	979 (37.3)	162 (36.3)				
cN1	1109 (42.3)	185 (41.5)				
cN2-3	536 (20.4)	99 (22.2)				
ER			39.833	0.000 *	0.713 (0.636–0.798)	0.000 *
0–9%	167 (6.4)	58 (13.0)				
10–49%	129 (4.9)	36 (8.1)				
50–89%	937 (35.7)	173 (38.8)				
90–100%	1391 (53.0)	179 (40.1)				
PR			37.208	0.000 *	0.644 (0.515–0.806)	0.000 *
Negativity	624 (23.8)	167 (37.4)				
Positivity	2000 (76.2)	279 (62.6)				
HER2			5.759	0.016 *	0.800 (0.635–1.009)	0.059
Zero	604 (23.0)	126 (28.3)				
Low	2020 (77.0)	320 (71.7)				
Ki67			46.900	0.000 *	0.490 (0.394–0.608)	0.000 *
≤14%	1153 (43.9)	274 (61.4)				
>14%	1471 (56.1)	172 (38.6)				
P53			3.526	0.060	-	-
Negative	1070 (40.8)	203 (45.5)				
Positive	1554 (59.2)	243 (54.5)				

* indicates statistically significant results.

**Table 4:** Baseline patient characteristics stratified by PR status (PR negativity vs. PR positivity).

Variable	PR− (n = 791)	PR+ (n = 2279)	χ^2^	*p* Value *
Age			0.345	0.557
<50 years	442 (55.9)	1246 (54.7)		
≥50 years	349 (44.1)	1033 (45.3)		
Menopausal status			0.422	0.516
Pre/Peri-	480 (60.7)	1353 (59.4)		
Post-	311 (39.3)	926 (40.6)		
Family history			1.689	0.430
No	617 (78.0)	1826 (80.1)		
Breast	60 (7.6)	161 (7.1)		
Others	114 (14.4)	292 (12.8)		
ypT			34.178	0.000 *
ypT0	177 (22.4)	310 (13.6)		
ypT1	185 (23.4)	580 (25.4)		
ypT2	323 (40.8)	1030 (45.2)		
ypT3-4	106 (13.4)	359 (15.8)		
ypN			19.073	0.000 *
ypN0	282 (35.7)	641 (28.1)		
ypN1	203 (25.7)	579 (25.4)		
ypN2-3	306 (38.6)	1059 (46.5)		
ER			235.469	0.000 *
0–9%	146 (18.5)	79 (3.5)		
10–49%	72 (9.1)	93 (4.1)		
50–89%	248 (31.3)	862 (37.8)		
90–100%	325 (41.1)	1245 (54.6)		
HER-2			8.407	0.004 *
Zero	218 (27.6)	512 (22.5)		
Low	573 (72.4)	1767 (77.5)		
Ki67			18.035	0.000 *
≤14%	419 (53.0)	1008 (44.2)		
>14%	372 (47.0)	1271 (55.8)		
P53			34.386	0.000 *
Negative	398 (50.3)	875 (38.4)		
Positive	393 (49.7)	1404 (61.6)		
pCR (ypYT0/N0)			37.208	0.000 *
No	624 (78.9)	2000 (87.8)		
Yes	167 (21.1)	279 (12.2)		
Radiotherapy			0.420	0.517
No	237 (30.0)	711 (31.2)		
Yes	554 (70.0)	1568 (68.8)		
Endocrine therapy			0.013	0.909
AI ± OFS	610 (77.1)	1762 (77.3)		
SERM ± OFS	181 (22.9)	517 (22.7)		
Extranodal extension			8.260	0.004 *
No	554 (70.0)	1468 (64.4)		
Yes	237 (30.0)	811 (35.6)		
Lymphovascular Invasion			0.324	0.569
No	577 (72.9)	1686 (74.0)		
Yes	214 (27.1)	593 (26.0)		

* Indicates statistically significant results.

**Table 5:** Baseline patient characteristics stratified by HER2 status (HER2-zero vs. HER2-low).

Variable	Total (n = 3070)	HER2-0 (n = 730)	HER2-Low (n = 2340)	χ^2^	*p* Value *
Age				0.001	0.504
<50 years	1688 (55.0)	401 (54.9)	1287 (55.0)		
≥50 years	1382 (45.0)	329 (45.1)	1053 (45.0)		
Menopausal status				0.352	0.291
Pre/Peri-	1833 (59.7)	429 (58.8)	1404 (60.0)		
Post-	1237 (40.3)	301 (41.2)	936 (40.0)		
Family history				3.787	0.151
No	2443 (79.6)	568 (77.8)	1875 (80.1)		
Breast	221 (7.2)	50 (6.9)	171 (7.3)		
Others	406 (13.2)	112 (15.3)	294 (12.6)		
ypT				9.759	0.021 *
ypT0	487 (15.9)	141 (19.3)	346 (14.8)		
ypT1	765 (24.9)	174 (23.8)	591 (25.2)		
ypT2	1353 (44.1)	300 (41.1)	1053 (45.0)		
ypT3-4	465 (15.1)	115 (15.8)	350 (15.0)		
ypN				1.417	0.492
ypN0	923 (30.1)	229 (31.4)	694 (29.6)		
ypN1	782 (25.4)	190 (26.0)	592 (25.3)		
ypN2-3	1365 (44.5)	311 (42.6)	1054 (45.1)		
ER				17.301	0.001 *
0–9%	225 (7.3)	65 (8.9)	160 (6.8)		
10–49%	165 (5.4)	51 (7.0)	114 (4.9)		
50–89%	1110 (36.2)	223 (30.5)	887 (37.9)		
90–100%	1570 (51.1)	391 (53.6)	1179 (50.4)		
PR				8.407	0.004 *
Negativity	791 (25.8)	218 (29.9)	573 (24.5)		
Positivity	2279 (74.2)	512 (70.1)	1767 (75.5)		
Ki67				0.824	0.364
≤14%	1427 (46.5)	350 (47.9)	1077 (46.0)		
>14%	1643 (53.5)	380 (52.1)	1263 (54.0)		
P53				15.873	0.000 *
Negative	1273 (41.5)	349 (47.8)	924 (39.5)		
Positive	1797 (58.5)	381 (52.2)	1416 (60.5)		
pCR (ypYT0/is, ypN0)				5.759	0.016 *
No	2624 (85.5)	604 (82.7)	2020 (86.3)		
Yes	446 (14.5)	126 (17.3)	320 (13.7)		
Radiotherapy				0.464	0.496
No	948 (30.9)	218 (29.9)	730 (31.2)		
Yes	2122 (69.1)	512 (70.1)	1610 (68.8)		
Endocrine therapy				2.013	0.156
AI ± OFS	2372 (77.3)	550 (75.3)	1822 (77.9)		
SERM ± OFS	698 (22.7)	180 (24.7)	518 (22.1)		
Extranodal extension				6.356	0.012 *
No	2022 (65.9)	509 (69.7)	1513 (64.7)		
Yes	1048 (34.1)	221 (30.3)	827 (35.3)		
Lymphovascular Invasion				13.318	0.000 *
No	2263 (73.7)	576 (78.9)	1687 (72.1)		
Yes	807 (26.3)	154 (21.1)	653 (27.9)		

* Indicates statistically significant results.

**Table 6:** Binary logistic regression analysis of HER2 status.

Variable	OR	95%CI	*p*
ypT	0.975	0.856–1.111	0.706
ER	1.327	1.197–1.472	0.000 *
PR	1.131	0.933–1.371	0.210
P53	1.381	1.164–1.637	0.000 *
Extranodal extension	1.233	1.020–1.489	0.030 *
pCR status	1.389	0.766–2.517	0.279
Lymphovascular Invasion	1.431	1.168–1.752	0.001 *

* Indicates statistically significant results.

## Data Availability

The data can be shared up on request.
